# Residential mobility and risk of childhood acute lymphoblastic leukaemia: an ecological study

**DOI:** 10.1038/sj.bjc.6603793

**Published:** 2007-05-29

**Authors:** A S Adelman, F D Groves, K O'Rourke, D Sinha, T C Hulsey, A B Lawson, D Wartenberg, D G Hoel

**Affiliations:** 1 Department of Biostatistics, Bioinformatics and Epidemiology, Medical University of South Carolina, 135 Cannon Street, Charleston, SC 29425, USA; 2 Department of Epidemiology and Population Health, School of Public Health and Information Sciences, University of Louisville, 555 South Floyd Street, Room #4053, Louisville, KY 40202, USA; 3 Lawton and Rhea Chiles Research Center, University of South Florida, 3111 E. Fletcher Avenue, Tampa, FL 33613-4660, USA; 4 Department of Pediatrics, Medical University of South Carolina, 135 Rutledge Avenue, PO Box 250561, Charleston, SC 29425, USA; 5 Department of Epidemiology and Biostatistics at Arnold School of Public Health, University of South Carolina, Columbia, SC 29208, USA; 6 Department of Environmental and Occupational Medicine, at Robert Wood Johnson Medical School, University of Medicine and Dentistry of New Jersey, 170 Frelinghuysen Road, Piscataway, NJ 08854-5635, USA

**Keywords:** childhood acute lymphoblastic leukaemia, urbanisation, population dynamics, socioeconomic status, poverty, sex, race

## Abstract

We conducted an ecological analysis of childhood acute lymphoblastic leukaemia-incidence data from children ⩽5 years old during 1992–1998 from the Surveillance, Epidemiology, and End Results Program in 200 counties and Hawaii. The response variable was the count of cases in each county race–sex stratum, examined in relation to data from the United States Census and the United States Department of Agriculture. The final models for both sexes included race, proportion moved during 1985–1990, and proportion of households with income ⩾$5000 as potential predictors. Incidence was lower among black boys (rate ratio (RR)=0.5) and black girls (RR=0.4) than among other children of the same sex; no other significant racial differences were detected. Incidence was elevated among males (but not females) residing in counties where ⩾50% of the population relocated (RR=1.5) and among females (but not males) residing in counties where <6% of the households had incomes <$5000 (RR=1.5). These sex differences in risk factors were unexpected.

A few studies of childhood acute lymphoblastic leukaemia (ALL) (Alexander *et al*, 1993; Stiller and Boyle, 1996; Infante-Rivard *et al*, 2000) have found associations with rural population growth or residential relocation (Kinlen *et al*, 1991; Alexander *et al*, 1997; Koushik *et al*, 2001). Studies of the relationships with urbanisation or population density have yielded inconsistent results. UK studies (Alexander *et al*, 1990b, 1996; Gilman and Knox, 1998; Dickinson and Parker, 1999) have found higher rates of childhood ALL or non-Hodgkin's lymphoma (NHL) in rural areas. However, studies elsewhere (McWhirter and Bacon, 1980; Muirhead, 1995; Petridou *et al*, 1997; Li *et al*, 1998; Hjalmars and Gustafsson, 1999; Adelman *et al*, 2005) have found higher ALL incidences in urban or high-density regions.

The relationship of socioeconomic status (SES) and childhood ALL is unclear. Some studies reported elevated childhood leukaemia rates in higher-SES areas (Kinlen *et al*, 1995; Stiller and Boyle, 1996; Borugian *et al*, 2005), whereas other studies have found no association (Alexander *et al*, 1990b; Kinlen *et al*, 1993; Dickinson and Parker, 1999).

This study aimed (1) to expand our understanding of the possible infectious nature of childhood ALL by examining its association with residential relocation, population change, and urbanisation, (2) to examine its relation with housing age (newly-constructed housing may reflect population growth or influx, but has not been previously studied), and (3) to clarify its relationship with SES by examining household income and the proportion of families below the poverty level.

## MATERIALS AND METHODS

Cancer registry data on childhood ALL incidences at ages 0–4 years in 1992–1998 were obtained from the Surveillance, Epidemiology, and End Results (SEER) Program of the National Cancer Institute. Incidence data as counts and person-years at risk (PYAR) were coded by sex and race (white/black/‘other’) for 200 individual counties covered by SEER in California, Connecticut, Georgia, Iowa, Michigan, New Mexico, Utah, and Washington, along with the state of Hawaii (which, for purposes of this study, will henceforth be considered a single county). Owing to its inordinately large size relative to other counties, both in terms of cases and PYAR, Los Angeles county was excluded.

For each county, six demographic variables were obtained from the 1990 and 2000 US censuses: residential relocation, population change, housing age, household income, and poverty. Urbanisation data were obtained from the United States Department of Agriculture (USDA), as described in greater detail below.

The 1990 census includes county-level data on numbers of people who changed their residence between 1985 and 1990, along with a detailed enumeration of those who previously resided in another country, state, county in the same state, or elsewhere in the same county. Thus, for each county the proportions of residents who moved – specifically from outside the country, the state, or the county, and total – were included in the analysis. These proportions relate to the whole 1990 census population of the counties.

### Population change, housing age, and household income

From the census data for 1990 and 2000, we derived the absolute and proportional change in population in each county. Data were obtained from the 2000 US census on the proportion of housing in each county constructed in each of the following periods: pre-1940, 1940–1949, 1950–1959, 1960–1969, 1970–1979, 1980–1989, 1990–1994, and post-1994. From these the proportion of housing units built since a range of cut-off points was calculated, most relevant to the years of incidence covered was 1990. The median year of construction was also available for homes in each county. Median household income for each county in 1989, which is available from the 1990 US census, was examined as a potential independent variable. In addition, the US Census Bureau breaks household income down into specific strata, for example, number of households with income <$5000, number of households with income $5000–$9999, number of households with income $10 000–$12 499, etc. This made it possible for us to calculate proportions of households whose income is above or below each of 24 distinct cut-off points.

### Poverty and urbanisation

Another SES variable for which data were available from the 1990 US census is the number of households below the poverty level in 1989, from which the proportion of households below the poverty level in each county was calculated. The degree of urbanisation of each county was categorised according to the 10 levels (0–9) of the ‘rural–urban continuum code’ developed by the USDA (Beale, 2003). Counties were first designated as metropolitan or nonmetropolitan. Metropolitan counties were further stratified by population size (⩾ or <1 000 000 people). The nonmetropolitan counties were divided into subgroups based on adjacency to metropolitan areas as well as percentage of population residing in urban areas. For comparability to a study published previously (Adelman *et al*, 2005) and to obtain reasonably precise point estimates of incidence rates, we opted to emphasise the proximity of nonmetropolitan counties to metropolitan areas while collapsing the subcategories of urban population among the nonmetropolitan counties. Counties in this study were grouped into four strata in order of decreasing urbanisation: metropolitan counties with population ⩾1 000 000 (codes 0 and 1), metropolitan counties with population <1 000 000 (codes 2 and 3), nonmetropolitan counties, adjacent to a metropolitan area (codes 4, 6, and 8), and nonmetropolitan counties, not adjacent to a metropolitan area (codes 5, 7, and 9).

### Modelling

The data were analysed using Poisson regression (Neter *et al*, 1996) in *R* (R Development Core Team, 2006; Venables *et al*, 2006), with each variable entered first in a single-variable (unadjusted) model. Population change was entered in two ways: as the absolute increase or decrease and as the proportionate increase or decrease. All other variables, except for race and sex, were modelled as a proportion of the total county population and log-transformed, reversing the signs to avoid entering values less than 0. The natural logarithm of the PYAR was used as an offset. Quadratic and cubic models for certain variables were calculated when scatter plots suggested that a polynomial model would fit the observed data better.

A set of variables was chosen which:

1. yielded statistically significant results in the univariate models,
2. were not highly correlated, and
3. fit better than other, related variables (e.g. although both total proportion moved and proportion moved from outside county were statistically significantly associated with childhood ALL among males, only total proportion moved was used, because it fit the data better.)

These variables and all possible two-way interactions among them were included in an initial model with multiple explanatory variables. The multivariate models had the form: 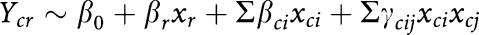 where *Y*_*cr*_ is the rate of childhood ALL for some stratum for race *r* of county *c*, *x*_*r*_ is a Boolean which is true when the county–race–sex stratum is of race *r*, *x*_*ci*_ is the value of the explanatory variable being examined for county c for some explanatory variable *i* (other than race), and every *β* and *γ* is a coefficient, each *γ* specifically for an interaction term. (The data are arranged such that there are separate values of observed cases and PYAR for each combination of race and sex in each county.) For both sexes, the variables used were race (black *vs* non-black; ‘other’ race was insignificant), proportion moved (linear, quadratic, and cubic terms), and proportion of households with income ⩾$5000. Successive multivariate models were then constructed for each sex by creating a new set of models, each of which lacked one of the variables of the original model, and choosing the model with the smallest deviance and of which the fit was not statistically and significantly worse (judged by the likelihood ratio test) than the original model; the chosen model became the new ‘original’ model and the cycle repeated until all derived models were worse than the ‘original’ model (essentially this is a variant on backwards step-wise selection). Finally, any interactions still present, which were based on small numbers, were dropped.

To assist interpretation, effects of continuous variables (linear, quadratic, or cubic terms) have not been tabulated but instead recategorised into strata in the results (certain results will not make sense unless this is borne in mind). For the variables not included in the final, multivariate model, quartiles weighted by PYAR were used; variables incorporated in the final multivariate model were rendered dichotomous.

## RESULTS

Results for variables included in the multivariate models are given in Table 1. The ALL incidence rates per 100 000 PYAR were 7.1 for white males (*n*=382), 6.8 for ‘other’ males (*n*=58), 3.5 for black males (*n*=32), 5.8 for both white females (*n*=300) and ‘other’ females (*n*=47), and 1.9 for black females (*n*=17), with a total of 836 cases in 1992–1998. Incidence of childhood ALL was statistically significantly lower for blacks than whites (rate ratio (RR)=0.5 for males, 0.4 for females); rates for ‘others’ did not differ significantly from those for whites.

### Residential relocation

The proportion of county residents who relocated any distance between 1985 and 1990 (‘proportion moved’ for short) ranged from 23 to 62%, who relocated from outside the county from 9 to 46%, who relocated from outside the state from 2 to 34%, and from outside the country from 0 to 9%.

A cubic model for proportion moved provided the best fit for both sexes combined, with the highest incidence of childhood ALL occurring in counties in which more than 50% of the residents had relocated between 1985 and 1990 (RR=1.2); this RR does not reflect sex-specific RRs. However, the corresponding cubic model for males provided the best fit and was statistically significant (RR=1.5). Among females, the cubic model for proportion moved was not statistically significant, although that of the quadratic model was (RR=0.9).

The linear and quadratic models for the proportion moved from outside the county were also significant for both sexes combined, with RRs of 1.2, 1.4, and 1.2 for the second, third, and fourth quartiles, respectively. Among males, only the linear model for the proportion moved from outside the county was significant, with RRs of 1.1, 1.5, and 1.4 for the second, third, and fourth quartiles, respectively (trend *P*<0.01). Among females, only the quadratic model for the proportion moved from outside the county was significant (RRs=1.2, 1.1, and 1.0 for the second, third, and fourth quartiles, respectively; trend *P*=0.73). The proportion moved from outside the state was not associated with childhood ALL among children of either sex.

### Population change, housing age, and household income

County populations in 1990 ranged from 690 to 2 112 000. The range of absolute population change between 1990 and 2000 was −50 530 to 235 500 and that of proportional population change during the same period was −20 to +90%.

Population in 1990 and proportional population change were not statistically significantly associated with childhood ALL incidence. Incidence was higher in rapidly growing counties, the RRs being 1.3, 1.5, and 1.3 for the second, third, and fourth quartiles of absolute population change (trend *P*<0.01), but this effect was statistically significant only among males, whose quartile-specific RRs were 1.5, 1.4, and 1.3, respectively (trend *P*=0.01).

The proportions of homes built since 1990 and 1995 were not associated with childhood ALL incidence among either males or females, nor was the median year of construction; however, counties with higher proportions of homes constructed since 1940, 1950, 1960, 1970, and 1980 tended to have elevated ALL incidence. None of the age-of-housing variables could be included in our multivariate model owing to their high degree of correlation with the proportion moved, which made multicolinearity a concern (*R* ranging from 0.46 to 0.70 except for median year structure built, which had *R*=−0.66).

The best-fitting variable in the household income category overall and among both sexes was the proportion of households in each county with incomes over $5000, which ranged from 80 to 99%; the RRs for the higher stratum of this variable were 1.6 among both sexes combined, 1.2 among males, and 1.5 among females. Proportions of households with income greater than or equal to many other thresholds ranging from $10 000 to $20 000 among males and to $100 000 among females also proved statistically significant, but became less so as the cut-off point was increased (data not shown).

County-level median household income in 1989 ranged from $12 990 to $54 800. There was a direct association between median income and ALL incidence among both sexes combined, with RRs of 1.1, 1.4, and 1.4 for the second, third, and fourth quartiles, respectively (trend *P*<0.01); this was statistically significant only among females, whose quartile-specific RRs were 1.3, 1.7, and 1.7 (trend *P*<0.01).

In short, children in higher-income counties had a higher incidence of childhood ALL than children in lower-income counties.

### Poverty and urbanisation

Childhood ALL incidence in both sexes was associated with the proportion of families living below the poverty level (range: 0–40%). Among both sexes combined, the RRs for the second, third, and fourth quartiles were 0.9, 1.0, and 0.7 (trend *P*<0.01), but a clear and statistically significant inverse association between poverty and childhood ALL incidence was present only among females, with RRs of 1.0, 1.0, and 0.7 (trend *P*<0.01).

Of the 50 metropolitan counties, 25 had a population ⩾1 million and of the 150 nonmetropolitan counties, 60 were adjacent to metropolitan areas. Urbanisation according to the USDA classification was inversely associated with childhood ALL among both sexes combined, with RRs of 1.0, 0.9, and 0.7 (trend *P*=0.08) for small metropolitan counties, adjacent nonmetropolitan counties, and nonadjacent nonmetropolitan counties, respectively; this inverse association was statistically significant only among females, with corresponding RRs of 1.1, 0.6, and 0.5 (trend *P*=0.02). Urbanisation was not included in the final model owing to its high correlation with the proportion moved (*R*=0.52), which conferred a high risk of multicolinearity.

## DISCUSSION

Among both sexes, higher childhood ALL incidence was associated with higher proportions of families with incomes greater than or equal to $5000 and white or ‘other’ race. Among males, higher childhood ALL was also associated with higher levels of proportion moved.

The higher incidence of childhood ALL among males was expected (Greenberg and Shuster, 1985; Parkin *et al*, 1988; MacMahon, 1992; Rechavi *et al*, 1992). Except for our previous finding of a rural–urban gradient among white males (but not females) (Adelman *et al*, 2005), few published studies have examined the role of sex directly in conjunction with other risk factors for childhood ALL, as we have done, in part because most studies of risk factors for ALL have either matched on or adjusted for sex. One previous study examining birth weight as a risk factor found that both birth weight over 3000 g and male gender were risk factors for childhood ALL in a multivariate model (Paltiel *et al*, 2004).

The lower incidence among blacks is well known (Greenberg and Shuster, 1985; Parkin *et al*, 1988; Rechavi *et al*, 1992; Smith *et al*, 1998, 1999; Adelman *et al*, 2005) and is conceivably due in part to socioeconomic disparities within the US. However, our data set cannot test this possibility and the ratio between the highest and lowest rates of the quartiles for income ⩾$5000 (an SES measure) was not large enough to account for race completely.

Childhood ALL was associated with residential relocation – total, from outside the county, and from outside the country, the latter among males only. Previous studies have found associations for childhood ALL with residential relocation (Alexander *et al*, 1993; Stiller and Boyle, 1996; Infante-Rivard *et al*, 2000) and the related variable rural population growth (Kinlen *et al*, 1991; Alexander *et al*, 1997; Koushik *et al*, 2001). This is consistent with an infectious aetiology, as children who move more would be expected to have more opportunities for exposure. However, the curvilinear relationships between residential relocation variables and childhood ALL incidence have not been reported previously. However, one could argue that since the hypothetical pathogen can spread only to previously unexposed children, the rate of childhood ALL may begin to decrease when the proportion of incomers exceeds half the population of the county.

We expected that new homes would be built in counties experiencing population influx, thereby producing a higher proportion of recently constructed housing units. We found that the proportions of housing units constructed since 1940, 1950, 1960, 1970, and 1980 were associated with increased incidence of ALL, but later cut-off points (1990 and 1995) were not; thus, our findings seem not to be driven by recent population influx.

Counties with a high proportion of households having a total income ⩾$5000 tended to have increased incidence of childhood ALL among both sexes; conversely, counties with a high proportion of households living below the poverty level tended to have decreased incidence of ALL. The proportion of county families living below the poverty level was inversely associated with incidence of ALL among females. Studies of SES and childhood ALL have been inconsistent, with elevated rates in higher-SES areas found by some (Pinkel and Nefzger, 1959; Fasal *et al*, 1971; Sanders *et al*, 1981; McWhirter, 1982; Laval *et al*, 1988; Cook-Mozaffari *et al*, 1989; Alexander *et al*, 1990a; Kinlen *et al*, 1995; Stiller and Boyle, 1996; Dockerty *et al*, 2001; Borugian *et al*, 2005), but not others (Alexander *et al*, 1990b; Kinlen *et al*, 1993; Dickinson and Parker, 1999). Our findings for household income and proportion of households below the poverty level better fit the former group, though from the shape of the curves, it appears that the issue is abject poverty rather the continuum of SES *per se*.

Urbanisation was associated with ALL only among females, whereas previous studies of urbanisation and population density have been inconsistent. In the United Kingdom (Alexander *et al*, 1990b, 1996; Gilman and Knox, 1998; Dickinson and Parker, 1999), higher rates of ALL or NHL in certain rural areas have been reported. However, in Taiwan (Li *et al*, 1998), Australia (McWhirter and Bacon, 1980), Greece (Petridou *et al*, 1997), Sweden (Hjalmars and Gustafsson, 1999), and the United States (Muirhead, 1995; Adelman *et al*, 2005), a higher incidence of ALL in urban or high-density regions has been found. We previously found high rates in the most urbanised areas, but there the rural–urban gradient was among white males but not among females (Adelman *et al*, 2005), whereas in this study urbanisation was not associated with childhood ALL among males.

In conclusion, childhood ALL was more common among whites than blacks and among males than females and was associated most strongly with household income and residential relocation. Our results for residential relocation are consistent with an infectious aetiology, but the curvilinear relationships between childhood ALL and residential relocation and the sex-specific responses were unexpected.

## Figures and Tables

**Table 1:** Variables included in the final, multivariate models

**Sex**	**Variable**	**Race–sex–county strata**	**Cases**	**PYAR**	**Unadjusted RR (95% CI), *P*-value**	**Adjusted RR (95% CI), *P*-value**
Both	Race					
	White	400	682	10 578 753	1^a^	1^a^
	Other	375	105	1 663 076	1.0 (0.8, 1.2), *P*=0.84	
	Black	285	49	1 787 199	0.4 (0.3, 0.6), *P*<0.01	0.5 (0.3, 0.6), *P*<0.01
						
	Proportion moved					
	(23, 50%)	842	380	7 175 672	1^a^	1^a^
	(50, 62%)	218	456	6 853 356	1.3 (1.1, 1.4), *P*<0.01	1.2 (1.1, 1.4), *P*<0.01
						
	Proportion of households with income ⩾$5000					
	(80, 94%)	446	107	2 708 359	1^a^	1^a^
	(94, 99%)	614	729	11 320 669	1.6 (1.3, 2.0), *P*<0.01	1.4 (1.1, 1.7), *P*<0.01
						
Females	Race					
	White	200	300	5 213 736	1^a^	1^a^
	Other	188	47	815 706	1.1 (0.8, 1.5), *P*=0.51	
	Black	146	17	880 211	0.3 (0.2, 0.6), *P*<0.01	0.4 (0.2, 0.6), *P*<0.01
						
	Proportion moved					
	(23, 50%)	425	187	3 512 466	1^a^	1^a^
	(50, 62%)	109	177	3 397 187	1.0 (0.8, 1.2), *P*=0.84	0.9 (0.8, 1.2), *P*=0.54
						
	Proportion of households with income ⩾$5000					
	(80, 94%)	227	46	1 389 972	1^a^	1^a^
	(94, 99%)	307	318	5 519 681	1.7 (1.3, 2.4), *P*<0.01	1.5 (1.1, 2.1), *P*<0.01
						
Males	Race					
	White	200	382	5 365 017	1^a^	1^a^
	Other	187	58	847 370	1.0 (0.8, 1.4), *P*=0.80	
	Black	139	32	906 988	0.5 (0.4, 0.7), *P*<0.01	0.5 (0.4, 0.8), *P*<0.01
						
	Proportion moved					
	(23, 50%)	417	193	3 663 206	1^a^	1^a^
	(50, 62%)	109	279	3 456 169	1.5 (1.3, 1.8), *P*<0.01	1.5 (1.2, 1.8), *P*<0.01
						
	Proportion of households with income ⩾$5000					
	(80, 94%)	219	61	1 318 387	1^a^	1^a^
	(94, 99%)	307	411	5 800 988	1.5 (1.2, 2.0), *P*<0.01	1.2 (0.9, 1.6), *P*=0.20

Abbreviations: PYAR=person years at risk; RR=rate ratio.

a Referent.
